# Stability Analysis of Unsteady Hybrid Nanofluid Flow over the Falkner-Skan Wedge

**DOI:** 10.3390/nano12101771

**Published:** 2022-05-23

**Authors:** Nurul Amira Zainal, Roslinda Nazar, Kohilavani Naganthran, Ioan Pop

**Affiliations:** 1 Department of Mathematical Sciences, Faculty of Science and Technology, Universiti Kebangsaan Malaysia, Bangi 43600, Malaysia; nurulamira@utem.edu.my (N.A.Z.); rmn@ukm.edu.my (R.N.); 2 Fakulti Teknologi Kejuruteraan Mekanikal dan Pembuatan, Universiti Teknikal Malaysia Melaka, Hang Tuah Jaya, Durian Tunggal 76100, Malaysia; 3 Institute of Mathematical Sciences, Faculty of Science, Universiti Malaya, Kuala Lumpur 50603, Malaysia; kohi@um.edu.my; 4 Center for Data Analytics, Consultancy and Services, Faculty of Science, Universiti Malaya, Kuala Lumpur 50603, Malaysia; 5 Department of Mathematics, Babeş-Bolyai University, 400084 Cluj-Napoca, Romania; 6 Academy of Romanian Scientists, 3 IIfov Street, 050044 Bucharest, Romania

**Keywords:** stability analysis, hybrid nanofluid, unsteady flow, moving wedge

## Abstract

Numerous manufacturing processes, including the drawing of plastic films, have a major impact on mass transport. These functionalities necessitate the solution of the Falkner–Skan equation and some of its configurations when applied to various geometries and boundary conditions. Hence, the current paper discusses the impact of unsteady hybrid nanofluid flow on a moving Falkner–Skan wedge with a convective boundary condition. This problem is modeled by partial differential equations, which are then converted into ordinary (similar) differential equations using appropriate similarity transformations. The bvp4c technique in MATLAB solves these ordinary differential equations numerically. Since more than one solution is possible in this paper, stability analysis is conducted. Thus, it is found that only one stable solution is identified as reliable (physically realizable in practice). The skin friction coefficient and heat transfer rate, along with the velocity and temperature profile distributions, are examined to determine the values of several parameters. The findings reveal that dual-type nanoparticles and wedge angle parameters improve thermal efficiency. A lower value of the unsteadiness parameter reduces the efficiency of hybrid nanofluids in terms of heat transfer and skin friction coefficient, whereas increasing the Biot number of the working fluid does not affect the critical point in the current analysis.

## 1. Introduction

A smart fluid with outstanding thermal capability is desirable to fulfill industrial and technical demands. In 1955, Choi [1] published work on an innovative heat transfer fluid based on nanotechnology called a nanofluid. Nanofluids, which are a colloidal mixture of nanoparticles (1–100 nm) and a base liquid (nanoparticle fluid suspensions), are a new class of nanotechnology for heat transfer (see Buongiorno et al. [2]). Nanofluids may be polymers, metals, metal oxides, or other materials. The use of nanoparticles significantly enhances the thermophysical characteristics of conventional heat transfer fluid, hence increasing its heat transfer coefficient. Due to this widespread recognition, nanofluids are now extensively employed in a variety of industries, including automotive, electronics, solar energy, biomedical, and oil recovery industries (see Singh et al. [3] and Suvardhan et al. [4]). Due to the vast interest in discovering effective methods to improve the performance of heating devices, nanofluids play important roles and have great potential in a variety of thermal applications, such as in heat transfer devices, which are used in various sectors of the economy, fuel cells, microelectronics, pharmaceutical processes, engine cooling/vehicles, chillers, and domestic refrigerators (see Chamsa et al. [5]). It has been observed that adding a small fraction of nanoparticles enhances a fluid’s thermal conductivity.

A new class of working fluids formed by two solid materials dispersed in a traditional fluid has been developed and extensively studied over the past few years. These fluids are known as hybrid nanofluids and are able to increase thermal conductivity and improve heat transfer in heat exchangers, significantly. Xian et al. [6], Babu et al. [7], and Huminic and Huminic [8] reviewed recent investigations on the synthesis, thermophysical properties, heat transfer characteristics, hydrodynamic behavior, and fluid flow characteristics reported by researchers on different hybrid nanofluids. These review papers also outlined the applications and challenges associated with hybrid nanofluids, and some suggestions for the future scopes of research in this fruitful area were also included. This type of hybrid nanofluid is found in various areas, including heat transfer, mechanical heat sinks, plate heat exchangers, helical heat exchangers, etc. Suresh et al. [9] conducted an experimental study on hybrid nanofluid characterization. Meanwhile, Devi and Devi [10,11], using the Tiwari and Das nanofluid model [12], showed the influence of magnetic parameters on hybrid nanofluid heat transfer rates compared to conventional viscous fluids. Takabi and Salehi [13] proposed new correlations based on thermophysical properties while examining the heat transfer performance of hybrid nanofluids. Very good reviews of papers on hybrid nanofluids have been published by Babu et al. [7], Huminic and Huminic [8], Muneeshwaran et al. [14], Sidik et al. [15], Sarkar et al. [16] and in the books by Das et al. [17], Nield and Bejan [18], Shenoy et al. [19], and Merkin et al. [20].

Historically, the solution of the Falkner–Skan [21] equation exemplified the application of Prandtl’s boundary layer theory on steady laminar flows passing a stationary wedge. Numerous manufacturing processes, including metal spinning, plastic film drawing, metallic plate cooling, the dynamism of pharmaceutical procedures, pace technology, nuclear reactor cooling, and many more, have a major impact on mass transport. These functionalities necessitate the solution of the Falkner–Skan equation [21] and some of its configurations when applied to various geometries and boundary conditions. Following this, over the last few decades, there have been significant research interests in understanding the Falkner–Skan [21] equation. Analytical investigations of this problem have been aimed at providing unique results and finding exact, nearly exact, or approximate analytical solutions. Computational approaches include a spectrum of methods, ranging from traditional finite-difference and finite-element methods to applying neural networks (see Asaithambi [22]). The boundary layer and several of its variants are applied to different geometries and corresponding boundary conditions. Several interesting papers on nanofluids discussing different aspects of nanofluids can be found in [23,24,25,26,27,28].

Hartree [29] revisited the Falkner–Skan problem that captivated many researchers’ interest in boundary layer flow past a moving wedge with various impacts. Yacob et al. [30] performed numerical simulations of the same problem with both static and moving wedges with the influence of prescribed surface heat flux in a nanofluid. Kudenatti et al. [31] investigated the stability of an Ostwald-de Waele model over a wedge, while Zainal et al. [32] scrutinized the effect of activation energy and chemical reactions over a moving wedge with a hybrid nanofluid. They discovered that increasing the volume fraction of nanoparticles improves heat transfer efficiency, whereas the activation energy factor has the opposite effect. Recently, Hussain et al. [33] verified that increasing the Biot number for convective heat transfers improves the thermal boundary layer thickness in the presence of the suction parameter. It is important to realize that there are numerous references on nanofluids past a moving wedge in steady and unsteady flows; for instance, Dinarvand et al. [34], Waini et al. [35], Awaludin et al. [36], and Murad et al. [37].

According to Zainal et al. [38,39], the addition of time-dependent terms to the governing equations that illustrate the unusual behavior in the unsteady flow has increased the fluid motion configuration and boundary layer separation. Over the last few centuries, mathematicians have focused on identifying the behavior of unsteady boundary layer flows, including the wedge problem under various conditions. Singh et al. [40] inspected mixed convection boundary layer flow past a vertical wedge, while Alam et al. [41] and Ali et al. [42] explored the various effects of unsteady flow over a moving wedge and heat transfer. Dual solutions are discovered in their numerical investigations, prompting the recent analyses of solution stability. The problem of boundary-layer growth on a body that is suddenly started from rest in an infinite, incompressible, viscous fluid has been investigated by many authors. Comprehensive reviews of the literature on steady and unsteady boundary-layer analyses are presented in Azam [43], Azam et al. [44], Riley [45], Telionis [46,47], and Ludlow et al. [48]. However, fewer studies have been concerned with the heat transfer aspects (see [49,50,51]).

Boundary layer flows have been investigated, either using a constant surface temperature boundary condition or a constant heat flux boundary condition. The application of convective boundary conditions, particularly in the engineering field, including transpiration cooling and material drying, demonstrates the significance of this requirement in boundary layer flow regimes. Convective heat transfer is extremely important in procedures involving high temperatures; for example, in the case of gas turbines, nuclear power plants, thermal energy storage, and so on. Meanwhile, convective boundary conditions are more convenient in many industrial and engineering processes, such as material drying, transpiration cooling, and so forth. Because of the practical significance of convective boundary conditions, numerous scholars have investigated and published results concerning nanofluids on this topic (see Malik et al. [52]).

Aziz [53] was a pioneer that initially proposed convective boundary conditions in the Blasius flow. Khan et al. [54] presented the similarity solutions of Falkner–Skan boundary layer flow of a nanofluid over a wedge with the convective boundary condition. The work in [54] demonstrated that dimensionless heat transfer rate increases as convective parameters are increased. Following that, several researchers examined convective phenomena in hybrid nanofluid using this type of boundary condition, for example, Khashi’ie et al. [55], Zainal et al. [56], Waini et al. [57], and Anuar et al. [58].

Despite these comprehensive literature reviews, there is still a lack of studies focusing on the unsteady hybrid nanofluids flow over moving Falkner–Skan wedge flow considering the convective boundary condition. Therefore, the present work attempts to analyze the unsteady hybrid nanofluid transport phenomena over a moving Falkner–Skan wedge with the presence of a convective boundary condition. Using the similarity transformation, the governing PDEs are converted into ODEs and are hence solved via the bvp4c technique in the MATLAB platform. The nanofluid model developed by Tiwari and Das [12], and the new thermophysical characteristics proposed by Takabi and Salehi [13], are employed to elucidate governing equations by incorporating dual-type nanoparticles, alumina (Al_2_O_3_) and copper (Cu), as well as water (H_2_O), as the base fluid. Variations in the local skin friction, local Nusselt number, velocity profiles, and temperature distributions are depicted graphically for various governing parameters. Since multiple solutions were presented, a stability analysis was carried out to justify the physical relevance of those solutions.

## 2. Mathematical Model

**Figure 1:**
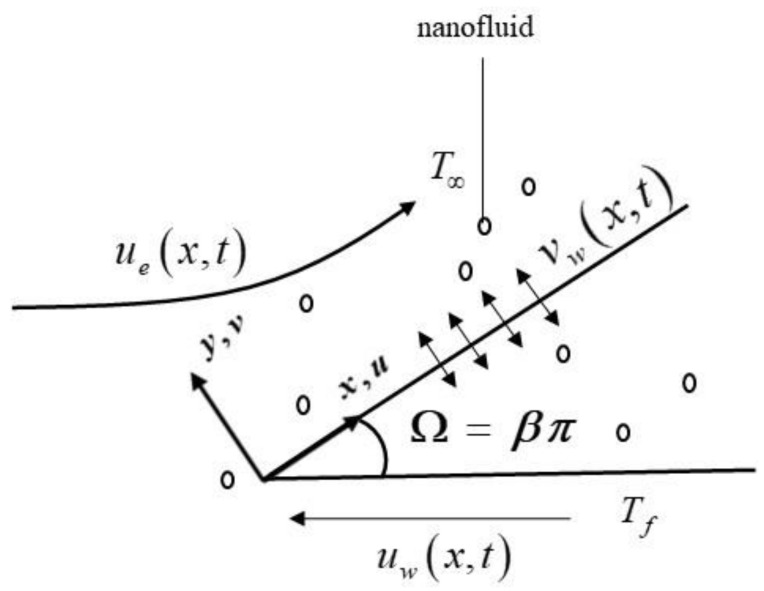
Physical model and coordinate system.

The present paper investigates the unsteady two-dimensional hybrid nanofluid over a moving Falkner–Skan wedge with a convective boundary condition. The Cartesian coordinates are denoted as x,y, where the x—axis is taken along the surface of the wedge, the y—axis is measured normal to it, and the flow is situated in the region of y≥0 (see Figure 1). We let the moving wedge velocity be $u_{w}\left(x,t\right)=U_{w}x^{m}/\left(1-\alpha t\right)$ and the far-field velocity be $u_{e}\left(x,t\right)=U_{e}x^{m}/\left(1-\alpha t\right)$, where α and $\left(U_{w},U_{e}\right)$ are constants with $U_{w}<0$ (moving wedge to the left) and $U_{w}=0$ corresponds to the static wedge. Here, m=β/2−β, where m is the wedge angle and β is the Hartree pressure gradient parameter, which corresponds to β=Ω/π for a total wedge angle Ω. Further, 0≤m≤1 where m=1 is the state of unsteady flow on a moving flat plate near the stagnation point and m=0 denotes the unsteady flow past a moving flat plate. Next, we assume that the upward surface of the wedge is heated by convection from a hot fluid at a constant temperature $T_{f}$, which provides a heat transfer coefficient ${\tilde{h}}_{f}\left(x\right)$, while $T_{\infty }$ is the constant temperature of the far flow (base fluid). For thermal enhancement, two different nanoparticles are considered, namely, Al_2_O_3_ and Cu suspended in the base fluid, H_2_O.

According to such interpretations, the corresponding problem is further modeled in the Cartesian coordinates $\left(x,y\right)$ as follows (see Murad et al. [37]; Ishak et al. [59]):

(1) $$\frac{\partial u}{\partial x}+\frac{\partial v}{\partial y}=0,$$

(2) $$\frac{\partial u}{\partial t}+u\frac{\partial u}{\partial x}+v\frac{\partial u}{\partial y}=\frac{\partial u_{e}}{\partial t}+u_{e}\frac{du_{e}}{dx}+\frac{\mu_{hnf}}{\rho_{hnf}}\frac{\partial^{2}u}{\partial y^{2}},$$

(3) $$\frac{\partial T}{\partial t}+u\frac{\partial T}{\partial x}+v\frac{\partial T}{\partial y}=\frac{k_{hnf}}{{\left(\rho C_{p}\right)}_{hnf}}\frac{\partial^{2}T}{\partial y^{2}},$$

with respect to

(4) $$\left.\begin{array}{l} u=\varepsilon u_{w}\left(x,t\right),\;\;v=v_{w}\left(x,t\right),\;\;-k_{hnf}\frac{\partial T}{\partial y}={\tilde{h}}_{f}\left(x\right)\left(T_{f}-T\right)\;\;\mathrm{at}\;\;y=0,\\ u\to u_{e}\left(x,t\right)\;,T\to T_{\infty }\;\mathrm{as}\;y\to \infty . \end{array}\right\}$$

From the above equations, the wedge surface velocity components are denoted as $\left(u,v\right)$, T is the fluid temperature, $\varepsilon =U_{w}/U_{e}$ is the wall velocity ratio $U_{w}$ towards $U_{e}$ which denotes the free stream velocity. It is worth noting that $\varepsilon \left(>0\right)$ relates directly to the condition when the wedge shifts in the opposite direction, whereas $\varepsilon \left(<0\right)$ implies the condition when the wedge travels parallel to the free stream, while ε=0 describes the static wedge. Note that $\mu_{hnf}$ and $k_{hnf}$ indicate the dynamic viscosity and heat conductivity, respectively, $\rho_{hnf}$ signifies density and ${\left(\rho C_{p}\right)}_{hnf}$ represents heat capacity. Table 1 shows characteristic properties used in this study (see Takabi and Salehi [13]) where ρ is density, $C_{p}$ and k indicate heat capacity constant pressure and thermal conductivity, respectively. The characteristic nanoparticle properties of Cu (copper) and Al_2_O_3_ (alumina), together with H_2_O (water), used as the base fluid (see Oztop and Abu Nada [60]), can be found in Table 2.

Here, ϕ is the nanoparticle volume fraction, where ϕ=0 corresponds to a regular fluid, $\phi_{1}$ denotes alumina nanoparticle (Al_2_O_3_), while $\phi_{2}$ represents copper nanoparticle (Cu). Based on the work done by Awaludin et al. [36] and Murad et al. [37], we introduce the appropriate transformations as follows:

(5) ψ=2Rexm+1fη, θη=T−TfTf−T∞, η=yx1+mRex2,

where $u=\frac{\partial \psi }{\partial y},\;v=-\frac{\partial \psi }{\partial x}.$

From the above transformations, Equation (1) is indeed satisfied. Hence, we obtained:

(6) u=uexm1−αtf′η, v=−12x2Rexm+1fη−ηf′η

Now, we take vwx,t=−12x2Rexm+1S, where $S=f\left(0\right)$ represents the constant mass flux, where S>0 and S<0 denote the fluid suction and injection, respectively. Next, for the governing equations, (1) to (3), to admit similarity solutions, we assume that h˜fx=hf/xRex1/2, where $h_{f}$ is a constant and Rex=uex,tx/νf is the local Reynolds number. In order to admit the similarity transformation, we also take $\tilde{A}=Al{\left[x/\left(1-\alpha t\right)x^{m}\right]}^{-1}$, where $A=\alpha l/U_{e}$ is a constant. With the help of the transformations (6), the governing equations, (2) and (3), reduce to the following ordinary differential (similarity) equations given by

(7) $$\frac{\mu_{hnf}/\mu_{f}}{\rho_{hnf}/\rho_{f}}f^{\prime \prime \prime }+ff^{\prime \prime }+\left(\frac{2m}{m+1}\right)\left(1-{f^{\prime }}^{2}\right)-\frac{\tilde{A}}{m+1}\left(2f^{\prime }+\eta f^{\prime \prime }-2\right)=0,$$

(8) $$\frac{1}{\mathrm{Pr}}\frac{k_{hnf}/k_{f}}{{\left(\rho C_{p}\right)}_{hnf}/{\left(\rho C_{p}\right)}_{f}}\theta^{\prime \prime }+f\theta^{\prime }-\left(\frac{4m}{m+1}\right)f^{\prime }\theta -\frac{\tilde{A}}{m+1}\left(4m\theta +\eta \theta^{\prime }\right)=0,$$

subject to the boundary conditions:

(9) $$\left.\begin{array}{l} f\left(0\right)=S,\;f^{\prime }\left(0\right)=\varepsilon ,\;-\sqrt{\frac{2m}{m+1}}\frac{k_{hnf}}{k_{f}}\theta^{\prime }\left(0\right)=\mathrm{Bi}\left[1-\theta \left(0\right)\right],\\ f^{\prime }\left(\eta \right)\to 1,\;\theta \left(\eta \right)\to 0,\;\mathrm{as}\;\eta \to \infty . \end{array}\right\}$$

Here, $\tilde{A}$ is the unsteadiness parameter, Pr is the Prandtl number, and Bi is the Biot number, which are defined by:

(10) $$\mathrm{Pr}=\frac{{\left(\rho C_{p}\right)}_{f}}{k_{f}},\;\mathrm{Bi}=\frac{h_{f}}{k_{f}}\sqrt{\frac{\alpha l^{2}}{\nu_{f}}}.$$

The physical quantities of interest are the skin friction coefficient $C_{f}$ and the local Nusselt number $Nu_{x},$ which are defined as:

(11) $$C_{fx}=\frac{\mu_{hnf}}{\rho_{f}u_{e}{}^{2}}{\left(\frac{\partial u}{\partial y}\right)}_{y=0},\;Nu_{x}=-\frac{xk_{hnf}}{k_{f}\left(T_{f}-T_{\infty }\right)}{\left(\frac{\partial T}{\partial y}\right)}_{y=0}$$

Using (6) and (11), we get:

(12) Rex1/2Cf=μhnfμfm+12f″0, Rex−1/2Nux=−khnfkfm+12θ′0,

where Rex=uex,t/νf is the local Reynolds number.

## 3. Analysis of Solution Stability

In general, the solutions to similarity equations are not distinctive for designated initial and boundary conditions due to non-linearity, geometric variability, or fluid mechanical characteristics. These can produce a bifurcation of the solution, leading to several solutions. Thus, this section presents the stability analysis technique to assess the dual solutions by evaluating the generated results’ reliability (see Merkin [62,63]). We introduce Γ, a dimensionless variable, as below:

(13) u=uexm1−αt∂f∂ηη,Γ, v=−12x2Rexm+1fη,Γ−η∂f∂ηη,Γ, θη,Γ=T−TfTf−T∞, η=yx1+mRex2, Γ=uexm−11−αtt.

Now, by utilizing Equation (13) and the unsteady flow of Equations (7) and (8) above, we have:

(14) $$\frac{\mu_{hnf}/\mu_{f}}{\rho_{hnf}/\rho_{f}}\frac{\partial^{3}f}{\partial \eta^{3}}+f\frac{\partial^{2}f}{\partial \eta^{2}}+\frac{2m}{m+1}\left[1-{\left(\frac{\partial f}{\partial \eta }\right)}^{2}\right]-\frac{\tilde{A}}{m+1}\left[2\frac{\partial f}{\partial \eta }+\eta \frac{\partial^{2}f}{\partial \eta^{2}}-2\right]-\left(1+\alpha \Gamma \right)\frac{\partial^{2}f}{\partial \eta \partial \Gamma }=0,$$

(15) $$\frac{1}{\mathrm{Pr}}\frac{k_{hnf}/k_{f}}{{\left(\rho C_{p}\right)}_{hnf}/{\left(\rho C_{p}\right)}_{f}}\frac{\partial^{2}\theta }{\partial \eta^{2}}+f\frac{\partial \theta }{\partial \eta }-\left(\frac{4m}{m+1}\right)\frac{\partial f}{\partial \eta }\theta -\frac{\tilde{A}}{m+1}\left(4m\theta +\eta \frac{\partial \theta }{\partial \eta }\right)-\left(1+\alpha \Gamma \right)\frac{\partial \theta }{\partial \Gamma }=0,$$

subject to the following conditions:

(16) $$\begin{array}{l} f\left(0,\Gamma \right)=S,\;\frac{\partial f}{\partial \eta }\left(0,\Gamma \right)=\varepsilon ,\;-\sqrt{\frac{2m}{m+1}}\frac{k_{hnf}}{k_{f}}\frac{\partial \theta }{\partial \eta }\left(0,\Gamma \right)=\mathrm{Bi}\left[1-\theta \left(0,\Gamma \right)\right],\\ \frac{\partial f}{\partial \eta }\left(\eta ,\Gamma \right)\to 1,\;\theta \left(\eta ,\Gamma \right)\to 0,\;\mathrm{as}\;\eta \to \infty . \end{array}$$

After that, the steady flow solutions are evaluated, where: $f\left(\eta \right)=f_{0}\left(\eta \right)\;\mathrm{and}\;\theta \left(\eta \right)=\theta_{0}\left(\eta \right)$

(17) $$\begin{array}{c} f\left(\eta ,\Gamma \right)=f_{0}\left(\eta \right)+e^{-\omega \;\Gamma }H\left(\eta \right),\\ \theta \left(\eta ,\Gamma \right)=\theta_{0}\left(\eta \right)+e^{-\omega \;\Gamma }I\left(\eta \right), \end{array}$$

is initiated in accordance with Weidman’s approach [64]. Next, Equation (17) is preserved to solve the eigenvalue problems of Equations (14) and (15). Based on Equation (17), $H\left(\eta \right)$ and $I\left(\eta \right)$ are relatively small for $f_{0}\left(\eta \right)\;\mathrm{and}\;\theta_{0}\left(\eta \right)$, whereas ω signifies the eigenvalue. Following that, we define the steady-state flow’s solutions $f_{0}\left(\eta \right)\;\mathrm{and}\;\theta_{0}\left(\eta \right)$, which were then completed by Γ→0. Substituting Equation (17) into Equations (14) and (15), the linearized eigenvalue problem’s solution is eventually determined as:

(18) $$\frac{\mu_{hnf}/\mu_{f}}{\rho_{hnf}/\rho_{f}}H^{\prime \prime \prime }+f_{0}H^{\prime \prime }+f_{0}^{\prime \prime }H+\frac{4m}{m+1}f_{0}{}^{\prime }H^{\prime }-\frac{\tilde{A}}{m+1}\left(2H^{\prime }+\eta H^{\prime \prime }\right)+\omega H^{\prime }=0,$$

(19) $$\frac{1}{\mathrm{Pr}}\left(\frac{k_{hnf}/k_{f}}{{\left(\rho C_{p}\right)}_{hnf}/{\left(\rho C_{p}\right)}_{f}}\right)I^{\prime \prime }+f_{0}I^{\prime }+\theta_{0}{}^{\prime }H-\frac{4m}{m+1}\left(f_{0}I^{\prime }+\theta_{0}H^{\prime }\right)-\frac{\tilde{A}}{m+1}\left(4mI+\eta I^{\prime }\right)+\omega I=0,$$

(20) $$\begin{array}{l} H\left(0\right)=0,\;H^{\prime }\left(0\right)=0,\;I^{\prime }\left(0\right)=-\sqrt{\frac{m+1}{2m}}\frac{k_{f}}{k_{hnf}}\mathrm{Bi}I\left(0\right),\\ H^{\prime }\left(\eta \right)\to 0,\;I\left(\eta \right)\to 0\;\mathrm{as}\;\eta \to \infty . \end{array}$$

Finally, by relaxing a boundary condition, possible eigenvalues can be generated when $H^{\prime }\left(\eta \right)\to 0$ as η→∞ in Equation (20) is substituted with $H^{\prime \prime }\left(0\right)=1$ (see Harris et al. [65]).

## 4. Results Interpretation

Equations (7) and (8) and boundary conditions (9) have been scrutinized via the bvp4c scheme numerically (see Shampine et al. [66]). The bvp4c solver is a finite difference algorithm that generates the three-stage Lobattao-IIIa formula. This well-known approach consists of a collocation formula that provides the polynomial at a C^−1^ continuous solution which is fourth-order accurate in the specific interval. Moreover, the bvp4c approach is more consistent than other solvers due to the convergence rate which is up to 10^−10^.

The effects of velocity profile $f^{\prime }(\eta ),$ temperature fields θ(η), coefficient of skin friction $f^{\prime \prime }\left(0\right)$, and the local Nusselt number $-\theta^{\prime }\left(0\right)$ have been established by assigning some values to the non-dimensional parameters. The numerical values of preferred non-dimensional parameters, such as the nanoparticles volume fraction, ϕ, angle of the wedge m, unsteadiness parameter $\tilde{A}$, and suction parameter S, are assumed to be constant throughout the study, and the results are provided in tables and figures. Table 3 displays the values of $f^{\prime \prime }\left(0\right)$ produced in this study when $\phi_{1}=\phi_{2}=\tilde{A}=\mathrm{Bi}=S=1.0,\mathrm{Pr}=0.73$ for assorted m values in comparison to those findings disclosed by Murad et al. [37], Ishak et al. [59], and Ullah et al. [67]. The generated results in Table 3 revealed excellent agreement with previous findings, confirming the precision of mathematical formulation in the current work. Since there are two possible solutions, the stability solution procedure is significant to the study. In general, the first solution is reliable because this solution reaches the far-field boundary standard. Even so, by conducting a solution stability analysis, the authors can confidently demonstrate the viable solutions. The smallest eigenvalue, $\omega_{1}$, uncovers numerical results properties in the analysis of the solution stability technique, as previously discussed in the preceding section. When the smallest eigenvalue is positive, the flow is defined as stable because the solutions fulfill the stabilizing criterion of permitting an initial decay. However, a contradictory result is obtained as the smallest eigenvalue turns out to be negative; hence the flow is noted as being unstable. Table 4 shows that the first solution generates positive values in the stability solutions, whereas the second solution yields negative values, indicating that it is unstable.

**Figure 2:**
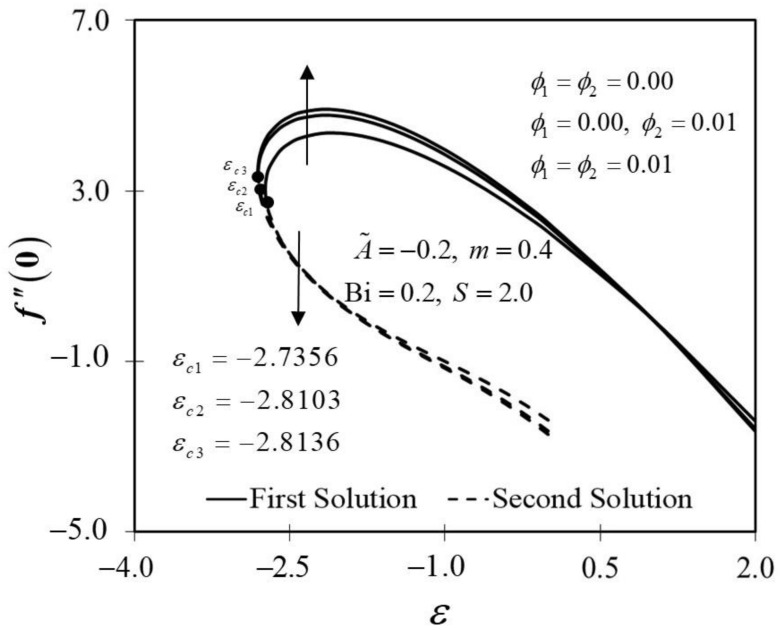
Trend of $f^{\prime \prime }\left(0\right)$ with ε and assorted ϕ.

**Figure 3:**
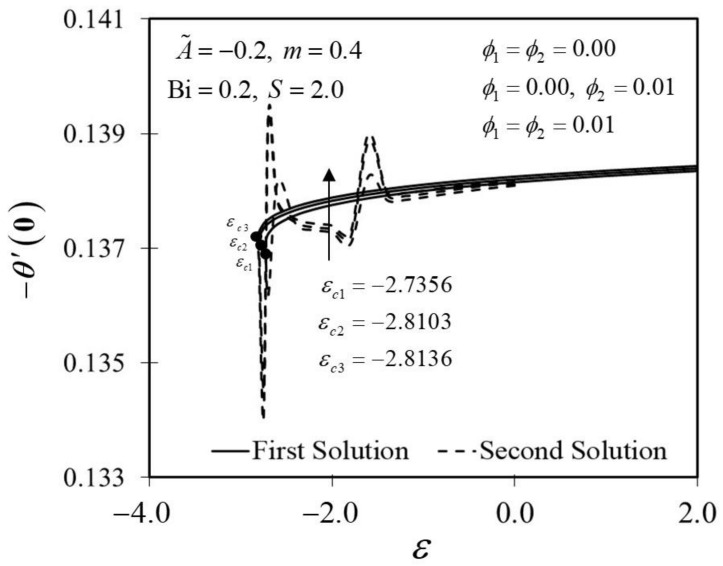
Trend of $-\theta^{\prime }\left(0\right)$ with ε and assorted ϕ.

**Figure 4:**
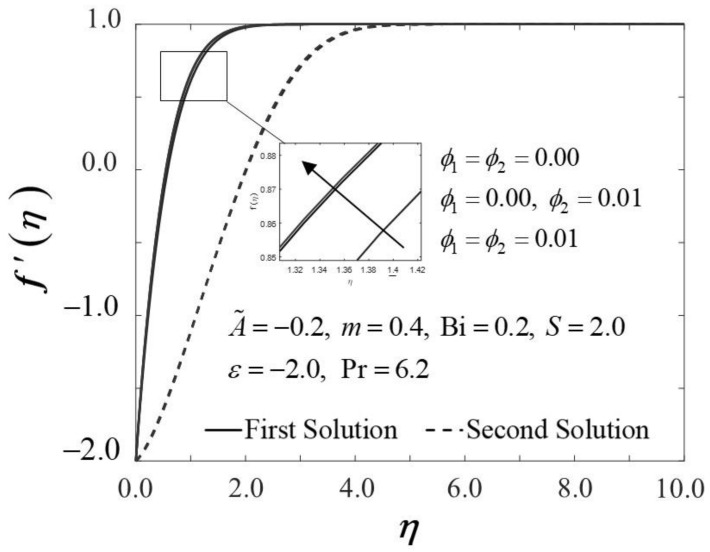
Trend of $f^{\prime }\left(\eta \right)$ with η and assorted ϕ.

**Figure 5:**
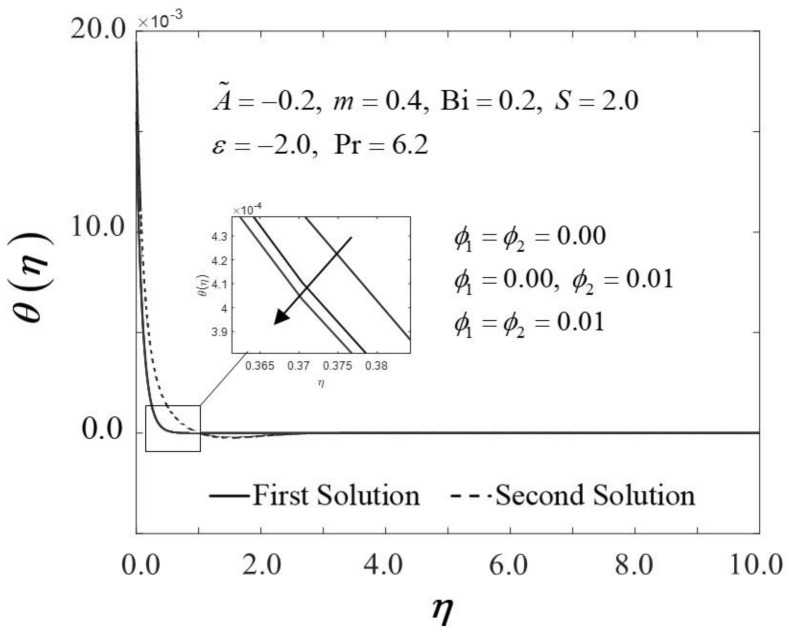
Trend of $\theta \left(\eta \right)$ with η and assorted ϕ.

Figure 2 and Figure 3 display the trend of $f^{\prime \prime }\left(0\right)$ and $-\theta^{\prime }\left(0\right)$ against ε with several ϕ, which represent several types of fluids, including viscous fluid $\left(\phi_{1}=\phi_{2}=0.00\right)$, alumina/water nanofluid $\left(\phi_{1}=0.00,\phi_{2}=0.01\right)$, and copper–alumina/water hybrid nanofluid $\left(\phi_{1}=\phi_{2}=0.01\right)$. As noted in Figure 2, the first solution tends to increase as ϕ increases, while the alternative solution displays an opposite behavior. Clearly, we can see that $f^{\prime \prime }\left(0\right)$ expands as the volume fraction of nanoparticles, ϕ, increases from a viscous fluid to a hybrid nanofluid in the first solution. When 1% and 2% of the total volume fraction of alumina is injected, the skin friction coefficient of the hybrid nanofluid and nanofluid is higher than the viscous fluid. The combination of nanoparticle volume concentration increased the working fluid’s viscosity; hence, the fluid velocity was boosted. The same characteristic is observed in Figure 3, which exposed the improvement in heat transfer rate $-\theta^{\prime }\left(0\right)$ towards the first solution as ϕ progresses. As a result, our findings support the notion that an increased concentration of nanoparticles in the working fluid helps to improve cooling efficiency as the viscous fluid transforms into a hybrid nanofluid. These findings are similar to the results obtained by Sarkar et al. [16]. According to their study, the synergistic effect in the nanoparticle can improve the heat transfer performance of a hybrid nanofluid. This has resulted in a heat transfer rate improvement with the addition of nanoparticle volume fractions. The impacts of ϕ on dimensionless $f^{\prime }(\eta )$ and θ(η) are shown in Figure 4 and Figure 5, respectively. The addition of ϕ accelerates fluid velocity as the wedge travels parallel to the free stream, as shown in Figure 4. Hence, it causes the fluid viscosity to decrease, developing the dimensionless velocity profile $f^{\prime }(\eta )$. On the other hand, Figure 5 depicts the nanoparticle volume fraction effect on the temperature profile θ(η) with assorted values of ϕ. The temperature distribution profile exhibits a downward trend in both solutions as ϕ inclines. Therefore, we can conclude that the inclusion of ϕ in hybrid nanofluids leads to a decrease in the boundary layer thickness; thus, the temperature distribution in the flow region declines as ϕ intensifies.

**Figure 6:**
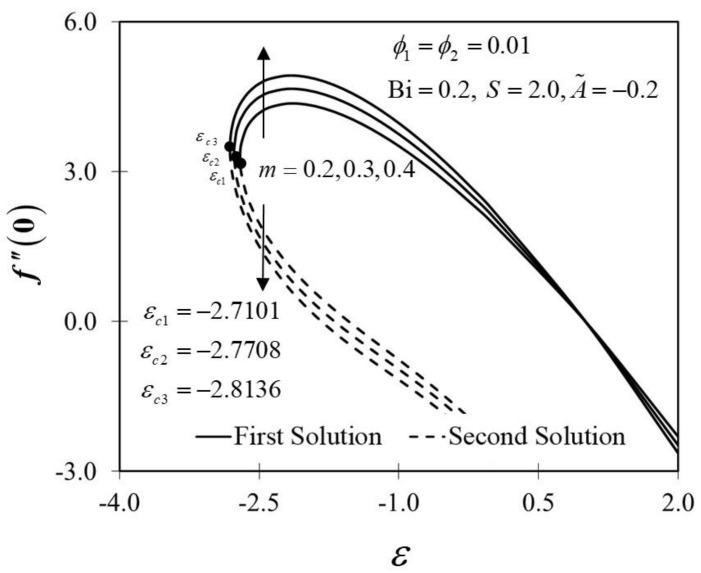
Trend of $f^{\prime \prime }\left(0\right)$ with ε and assorted m.

**Figure 7:**
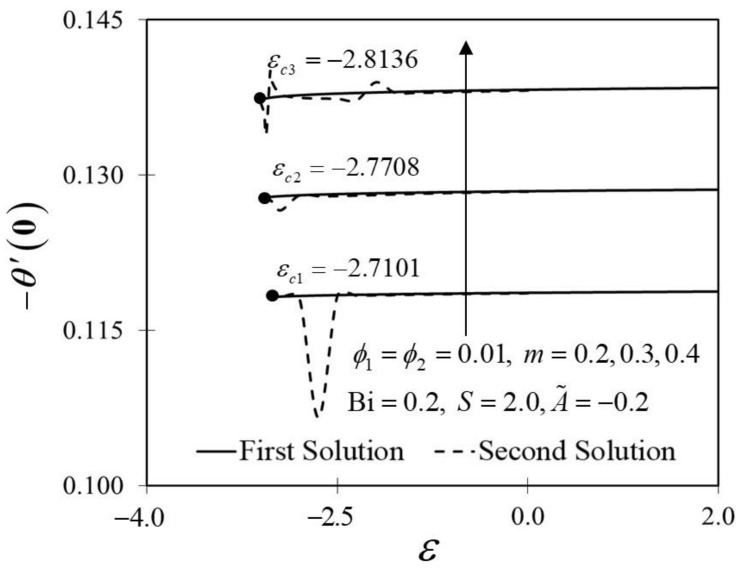
Trend of $-\theta^{\prime }\left(0\right)$ with ε and assorted m.

**Figure 8:**
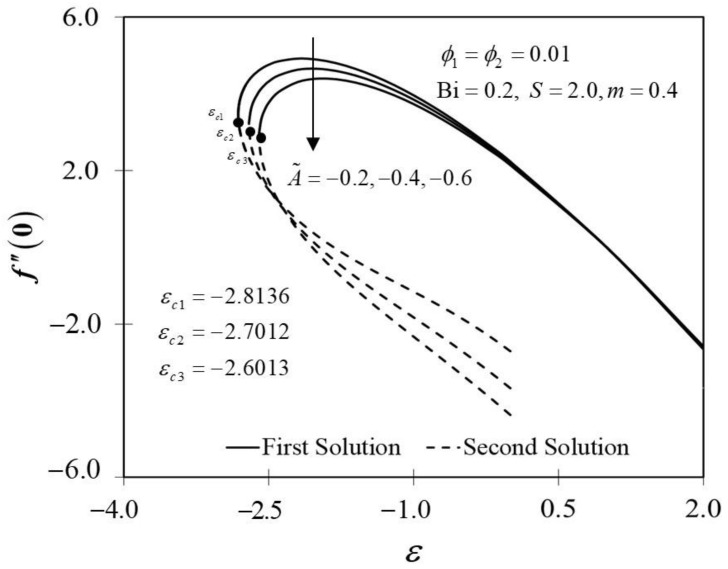
Trend of $f^{\prime \prime }\left(0\right)$ with ε and assorted A.

**Figure 9:**
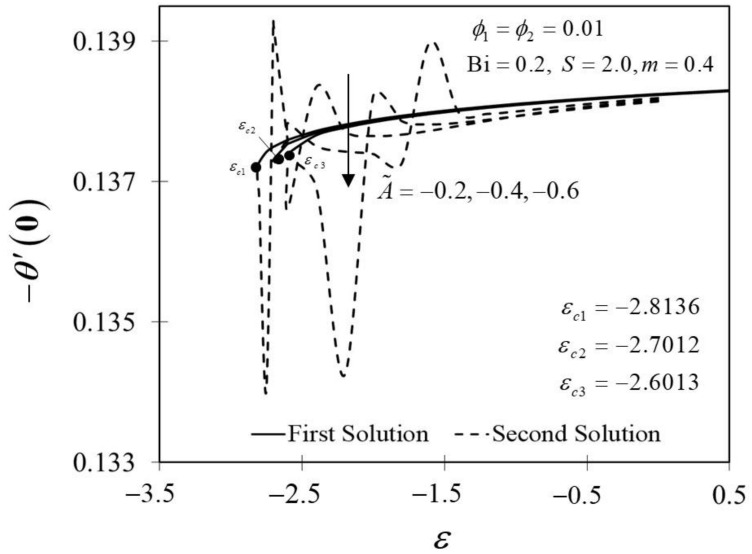
Trend of $-\theta^{\prime }\left(0\right)$ with ε and assorted A.

The significance of m on $f^{\prime \prime }\left(0\right)$ and $-\theta^{\prime }\left(0\right)$ against ε are depicted in Figure 6 and Figure 7. It is spotted that as m increases, $f^{\prime \prime }\left(0\right)$ and $-\theta^{\prime }\left(0\right)$ are plotted to be increased in the first solution. Similar results are observed in Waini et al. [35]. This appears to result in an increase of approximately 3.8% in heat transfer rate as m increased. Thus, higher values of m are proven to contribute to the working fluid’s thermal efficiency. In addition, Figure 6 also shows that when the wedge surface moves at a rate of ε=1.0, $f^{\prime \prime }\left(0\right)=0$. This explains the appearance of no frictional drag force on the progressed wedge surface, which is heated convectively. Furthermore, this study is also interested in examining the effect of the unsteadiness parameter, $\tilde{A}$ in hybrid nanofluid flow, which plays the role of heat transfer fluid, influencing mechanical behavior by observing how the coefficient of skin friction $f^{\prime \prime }\left(0\right)$ and heat transfer rate $-\theta^{\prime }\left(0\right)$ change. Figure 8 shows that the decrement of $\tilde{A}$ triggered $f^{\prime \prime }\left(0\right)$ to diminish in the first solution, and the reaction was in the opposite direction towards the second solution. While $\tilde{A}$ decreases, a reduction in the velocity gradient is observed, therefore $f^{\prime \prime }\left(0\right)$ diminishing. Subsequently, when $\tilde{A}$ is reduced, the obtained results of $-\theta^{\prime }\left(0\right)$ display a reduction trend in both solutions, as shown in Figure 9. This observation supports the idea that decreasing the unsteadiness strength lowers the heat transfer efficiency.

**Figure 10:**
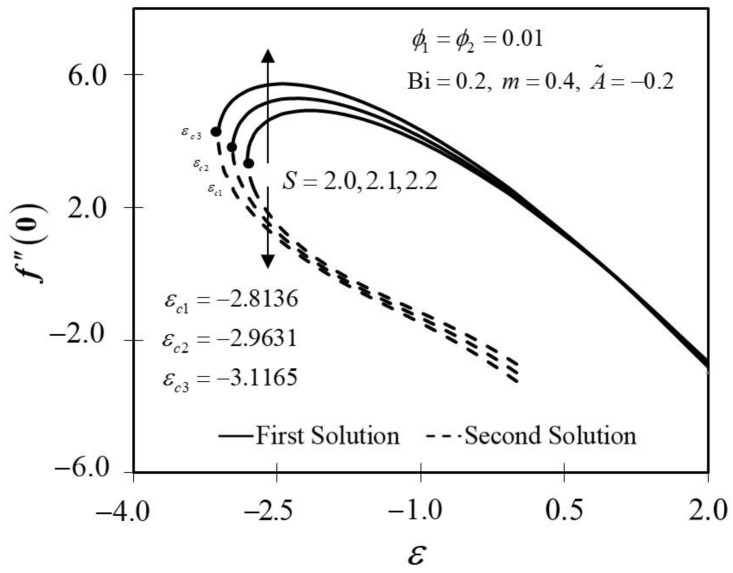
Trend of $f^{\prime \prime }\left(0\right)$ with ε and assorted S.

**Figure 11:**
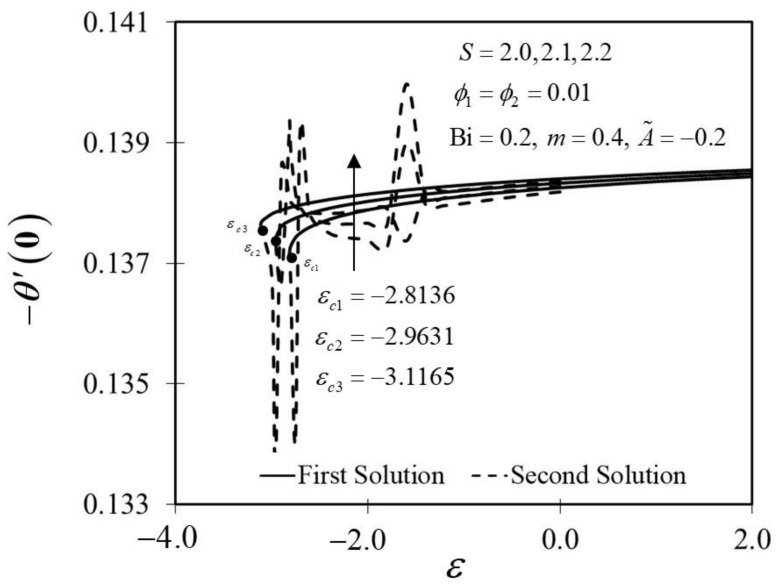
Trend of $-\theta^{\prime }\left(0\right)$ with ε and assorted S.

**Figure 12:**
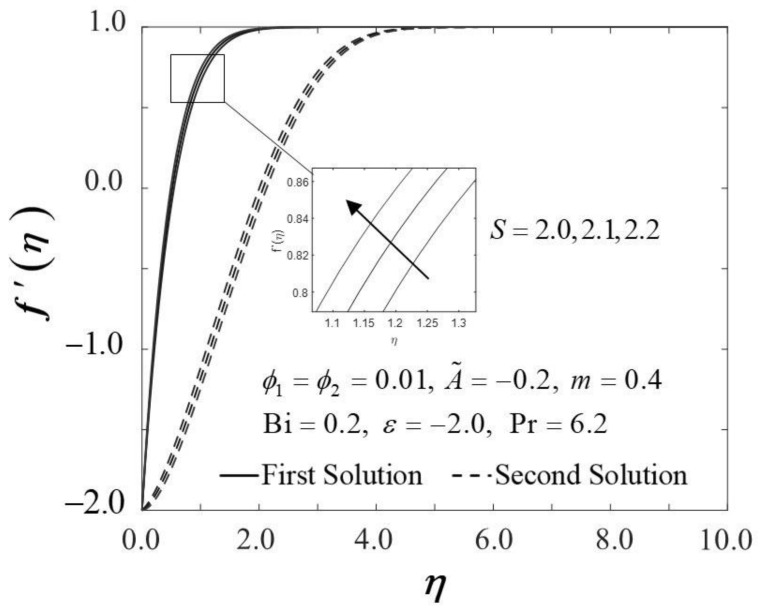
Trend of $f^{\prime }\left(\eta \right)$ with η and assorted S.

**Figure 13:**
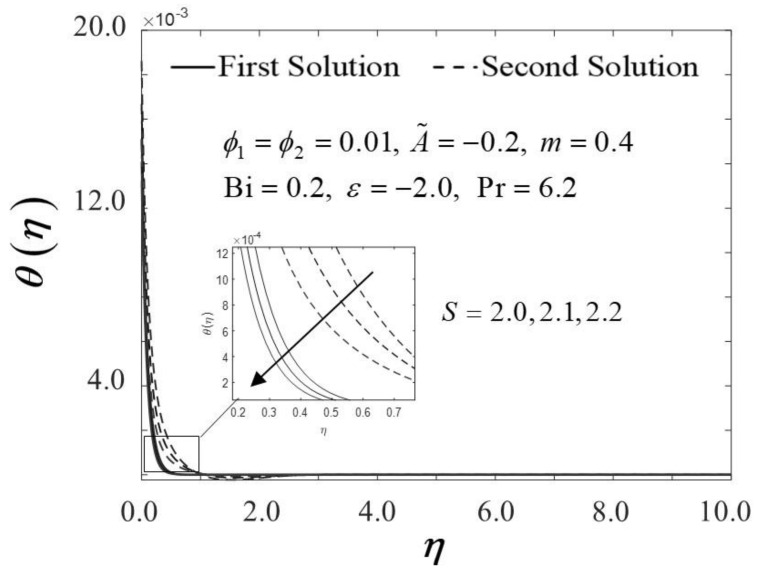
Trend of $\theta \left(\eta \right)$ with η and assorted S.

The consequence of the suction parameter S with ε is presented in Figure 10 and Figure 11. Figure 10 emphasizes that as S escalates, $f^{\prime \prime }\left(0\right)$ amplifies in the first solution of the hybrid nanofluid but not in the second solution. In addition, in this observation, the rising values of S expanded the dual solution domain ε, and caused an increment in the critical value $\left|\varepsilon_{c}\right|$ on the moving wedge of the hybrid nanofluid. This finding also contributes to the delay of the boundary layer separation process as S improves (see Figure 12). Additionally, the skin friction coefficient results recorded the highest level with the largest value of S in the hybrid nanofluid flow. Meanwhile, Figure 11 shows the value of $-\theta^{\prime }\left(0\right)$ increases with increasing value of S on the moving wedge surface in both solutions. This occurrence is caused by the increment of suction values, allowing the flow of hybrid nanofluid to approach the wedge surface, thus reducing the thickness of the thermal boundary layer (see Figure 13). As a result, the hybrid nanofluid flow travels at a high velocity, enhancing the shear stress and thus intensifying the thermal performance. In connection with the results discussed earlier in Figure 10 and Figure 11, Figure 12 depicts the influence of suction parameter on velocity profile $f^{\prime }\left(\eta \right)$, while Figure 13 shows the temperature field distributions $\theta \left(\eta \right)$ via implementing dual-type nanoparticles. The velocity profile inclines as the number of suction increases due to increased fluid viscosity. However, the presence of such particles tends to lower the temperature profile. This is due to a rise in the thermal conductivity of the mixing fluid, which improves heat transfer performance.

**Figure 14:**
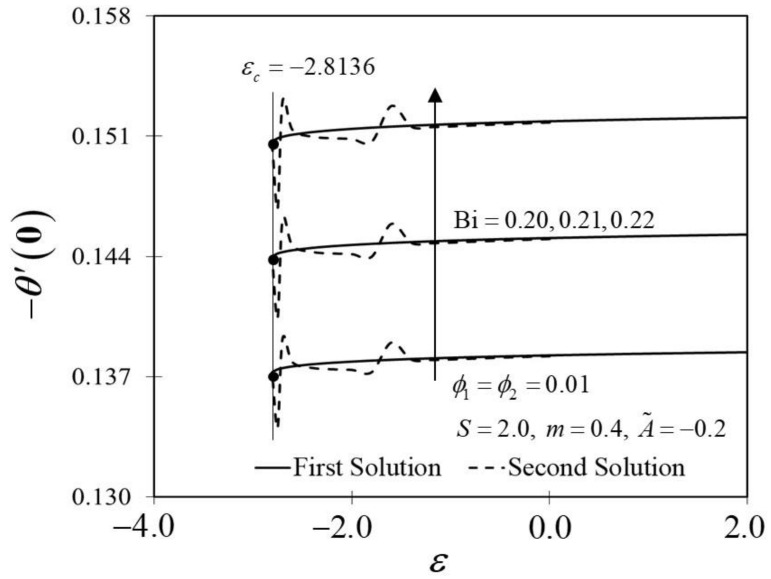
Trend of $-\theta^{\prime }\left(0\right)$ with ε and assorted Bi.

**Figure 15:**
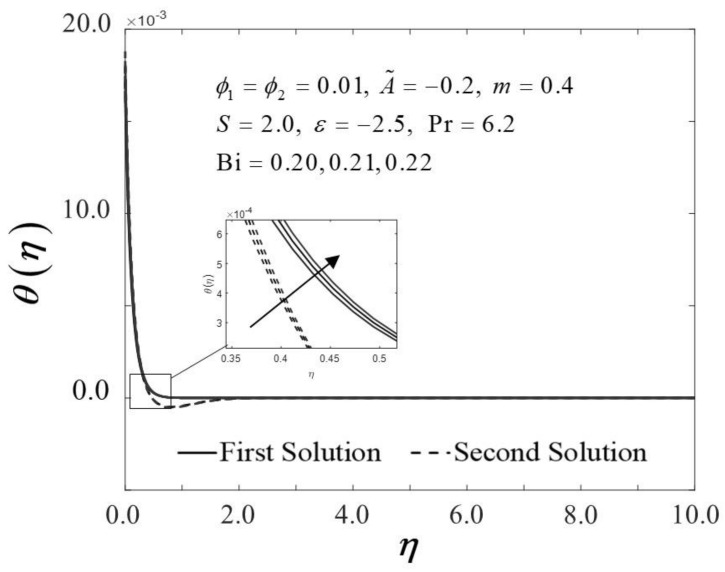
Trend of $\theta \left(\eta \right)$ with η and assorted Bi.

The Biot number (Bi) is described as the ratio of conduction to convection times. Since the small magnitude of Bi reduces the effect on high thermal regions, the range of Bi in this study is selected in between 0.20≤Bi≤0.22. Physically, enhancing the values of Bi, raises the temperature inside the boundary layer; however, it also creates a higher density thermal boundary layer and is effectively responsible for regulating the temperature within the thermal reactors. Figure 14 and Figure 15 show the result of various Bi on $-\theta^{\prime }\left(0\right)$ and thermal boundary layers $\theta \left(\eta \right)$, respectively. The improvement in $-\theta^{\prime }\left(0\right)$ is proven in Figure 14 as Bi increases towards the moving wedge, which significantly increases the rate of heat transfer with a similar value of critical point, i.e., $\varepsilon_{c}$. As predicted, higher surface temperatures arise from stronger convection, thus, enabling the thermal resistance to permeate deeper, as shown in Figure 15. The results are similar to those of Hussain et al. [33], who found that increasing the value of Bi for convective heat transfer improves the thermal boundary layer thickness.

## 5. Conclusions

Various effects of controlling parameters in the unsteady hybrid nanofluid flow and heat transfer on a moving Falkner–Skan wedge with a convective boundary condition are discussed in this numerical study. The physical properties of the fluid are affected by different values of the nanoparticle volume fraction, unsteadiness parameter, wedge angle parameter, suction parameter, and the Biot number. Using dual-type nanoparticles and increasing the suction parameter improves the velocity profile. Meanwhile, these two parameters also reduce the temperature field distributions, which improves the thermal system’s heat transfer effectiveness. On the other hand, it was discovered that decreasing the unsteadiness parameter caused nanoparticles to move slower, resulting in a decrease in flow speed, hence, significantly reducing the thermal efficiency. In contrast, the heat transfer is enhanced rapidly by improving the wedge angle parameter. Further, it is noted that the use of hybrid nanofluid, especially Al_2_O_3_-Cu/H_2_O, can be considered as a future heat transfer fluid in various heat transfer applications due to the effectiveness of the thermal performance when compared to the conventional fluid used in the current study. Finally, based on the stability analysis, the first solution is reliable, whereas the alternative solution is not.

## Figures and Tables

**Table 1:** The characteristic properties. (Takabi and Salehi [13]; Ghalambaz et al. [61]).

Characteristics	Alumina-Copper/Water (Al_2_O_3_–Cu/H_2_O)
Dynamic viscosity, $\mu_{hnf}$	$\mu_{hnf}/\mu_{f}={\left(1-\phi_{hnf}\right)}^{-2.5}$
$\mathrm{Heat}\;\mathrm{capacity},\;{\left(\rho C_{p}\right)}_{hnf}$	${\left(\rho C_{p}\right)}_{hnf}-\left(1-\phi_{hnf}\right){\left(\rho C_{p}\right)}_{f}=\phi_{1}{\left(\rho C_{p}\right)}_{Al_{2}O_{3}}+\phi_{2}{\left(\rho C_{p}\right)}_{Cu}$
$\mathrm{Density},\;\rho_{hnf}$	$\rho_{hnf}=\left(1-\phi_{hnf}\right)\rho_{f}+\phi_{1}\rho_{Al_{2}O_{3}}+\phi_{2}\rho_{Cu}$
$\mathrm{Heat}\;\mathrm{conductivity},\;k_{hnf}$	$\frac{k_{hnf}}{k_{f}}=\left[\frac{\left(\frac{\phi_{1}k_{Al_{2}O_{3}}+\phi_{2}k_{Cu}}{\phi_{hnf}}\right)+2k_{f}+2\left(\phi_{1}k_{Al_{2}O_{3}}+\phi_{2}k_{Cu}\right)-2\phi_{hnf}k_{f}}{\left(\frac{\phi_{1}k_{Al_{2}O_{3}}+\phi_{2}k_{Cu}}{\phi_{hnf}}\right)+2k_{f}-\left(\phi_{1}k_{Al_{2}O_{3}}+\phi_{2}k_{Cu}\right)+\phi_{hnf}k_{f}}\right]$

**Table 2:** The nanoparticles and base fluid properties. (see Oztop and Abu-Nada [60]).

Characteristics	$C_{p}\;\left(J/\mathrm{kgK}\right)$	k (W/mK)	$\rho \;({\mathrm{kg}/m}^{3})$
Cu	385	400	8933
Al_2_O_3_	765	40	3970
H_2_O	4179	21	0.613

**Table 3:** Results comparison of $f^{\prime \prime }\left(0\right)$ with different value of m while $\phi_{1}=\phi_{2}=\tilde{A}=\mathrm{Bi}=S=1.0\;\mathrm{and}\;\mathrm{Pr}=0.73$.

*m*	Ishak et al. [59]	Ullah et al. [67]	Murad et al. [37]	Present Result
	$f^{\prime \prime }\left(0\right)$			
0.0000	0.469750	0.469600	0.469000	0.4696000
0.0141	0.504720	0.504600	0.504620	0.5046143
0.0435	0.569040	0.569000	0.568980	0.5689778
0.0909	0.655010	0.655000	0.654980	0.6549789
0.1429	0.732020	0.732000	0.732000	0.7319986
0.2000	0.802140	0.802100	0.802130	0.8021256
0.3333	0.927660	0.927700	0.92766	0.9276536

**Table 4:** Results of the smallest eigenvalues $\omega_{1}$ by several ε.

ε	First Solution	Second Solution
−2.00	1.1634	−0.9750
−2.10	0.9497	−0.9221
−2.30	0.8344	−0.8871
−2.60	0.5774	−0.7898
−2.70	0.2463	−0.6132
−2.79	0.0056	−0.4339

## Data Availability

Not applicable.

## Nomenclature

*Roman letters*	
*A*	unsteadiness parameter $\left(-\right)$
Bi	Biot number $\left(-\right)$
$C_{f}$	skin friction coefficient $\left(-\right)$
$C_{p}$	specific heat at constant pressure $\left({\mathrm{Jkg}}^{-1}K^{-1}\right)$
${\tilde{h}}_{f}$	heat transfer coefficient $\left({\mathrm{Wm}}^{-2}K^{-2}\right)$
$f\left(\eta \right)$	dimensionless stream function $\left(-\right)$
k	thermal conductivity of the fluid $\left({\mathrm{Wm}}^{-1}K^{-1}\right)$
m	wedge angle parameter $\left(-\right)$
$Nu_{x}$	local Nusselt number $\left(-\right)$
$\left(pC_{p}\right)$	heat capacitance of the fluid $\left({\mathrm{JK}}^{-1}m^{-3}\right)$
Pr	Prandtl number $\left(-\right)$
Rex	local Reynolds number in x− axis $\left(-\right)$
S	constant mass flux $\left(-\right)$
t	time $\left(s\right)$
T	fluid temperature $\left(K\right)$
$T_{f}$	reference temperature $\left(K\right)$
$T_{\infty }$	ambient temperature $\left(K\right)$
u,v	velocities component in the x— and y— directions, respectively $\left({\mathrm{ms}}^{-1}\right)$
$u_{e}$	velocities of the far-field $\left({\mathrm{ms}}^{-1}\right)$
$u_{w}$	velocities of the moving wedge $\left({\mathrm{ms}}^{-1}\right)$
x,y	Cartesian coordinates $\left(m\right)$
*Greek symbols*	
α	constant
β	Hartree pressure gradient parameter $\left(-\right)$
ψ	stream function $\left(-\right)$
η	similarity variable $\left(-\right)$
θ	dimensionless temperature $\left(-\right)$
ε	wall velocity ratio $\left(-\right)$
μ	dynamic viscosity of the fluid $\left({\mathrm{kgm}}^{-1}s^{-1}\right)$
ν	kinematic viscosity of the fluid $\left(m^{2}s^{-1}\right)$
ρ	density of the fluid $\left({\mathrm{kgm}}^{-3}\right)$
Γ	dimensionless time variable $\left(-\right)$
$\phi_{1}$	nanoparticle volume fractions for Al_2_O_3_ (alumina) $\left(-\right)$
$\phi_{2}$	nanoparticle volume fractions for Cu (copper) $\left(-\right)$
ω	eigenvalue $\left(-\right)$
$\omega_{1}$	smallest eigenvalue $\left(-\right)$
*Subscripts*	
f	base fluid $\left(-\right)$
nf	nanofluid $\left(-\right)$
hnf	hybrid nanofluid $\left(-\right)$
s1	solid component for Al_2_O_3_ (alumina) $\left(-\right)$
s2	solid component for Cu (copper) $\left(-\right)$
*Superscript*	
${}^{\prime }$	differentiation with respect to η $\left(-\right)$
